# The Johns Hopkins Hospital Reports

**Published:** 1901-10

**Authors:** 


The Johns Hopkins Hospital Reports. Vol. VIII. Nos. 3–9. 1900.

This number of the Johns Hopkins Hospital Reports is devoted entirely to typhoid fever in its medical and surgical aspects, and is a most important addition to the literature of this disease.

Among the articles of exceptional interest are those of Dr. Osler, on hemiplegia in typhoid; chronic cystitis due to typhoid, by Dr. Young; Gall-bladder complications, by Dr. Camac; surgical treatment, by Dr. Finney; A study of the Widal reaction in 265 cases of typhoid, by Dr. Gwyn; coincident typhoid and malarial infection, by Dr. Irving P. Lyon, of Buffalo, formerly of Johns Hopkins; and other papers of much clinical value.

